# Identification of Major Flavone *C*-Glycosides and Their Optimized Extraction from *Cymbidium kanran* Using Deep Eutectic Solvents

**DOI:** 10.3390/molecules22112006

**Published:** 2017-11-18

**Authors:** Kyung Min Jeong, Misuk Yang, Yan Jin, Eun Mi Kim, Jaeyoung Ko, Jeongmi Lee

**Affiliations:** 1 School of Pharmacy, Sungkyunkwan University, Jangan-gu, Suwon 16419, Gyeonggi-do, Korea; aeroserp86@naver.com (K.M.J.); kimyeon909@gmail.com (Y.J.); em131632@gmail.com (E.M.K.); 2 Amorepacific Research and Development Center, Giheung-gu, Yongin 17074, Gyeonggi-do, Korea; msyang@amorepacific.com

**Keywords:** Orchidaceae, *Cymbidium*, flavone *C*-glycoside profile, vicenin-2, deep eutectic solvent, response surface methodology

## Abstract

*Cymbidium kanran*, an orchid exclusively distributed in Northeast Asia, has been highly valued as a decorative plant and traditional herbal medicine. Here, *C. kanran* extracts were prepared in 70% aqueous methanol using ultrasound-assisted extraction (UAE) and subjected to liquid chromatography-photodiode array detection and ultra-high performance liquid chromatography-quadrupole-time-of-flight-mass spectrometry analysis, which were used for quantitative and qualitative analysis, respectively. It was found that the extracts were rich in flavone *C*-glycosides including vicenin-2, vicenin-3, schaftoside, vitexin, and isovitexin. Ten deep eutectic solvents (DESs) were synthesized by combining choline chloride (hydrogen bond acceptor) with various polyols and diols (hydrogen bond donors) and were tested as a medium for the efficient production of extracts enriched with potentially bioactive flavone *C*-glycosides from *C. kanran*. A DES named ChCl:DPG, composed of choline chloride and dipropylene glycol at a 1:4 molar ratio, exhibited the best extraction yields. Then, the effects of extraction conditions on the extraction efficiency were investigated by response surface methodology. Lower water content in the extraction solvent and longer extraction time during UAE were desirable for higher extraction yields. Under the statistically optimized conditions, in which 100 mg of *C. kanran* powder were extracted in 0.53 mL of a mixture of ChCl:DPG and water (74:26, *w/w*) for 86 min, a total of 3.441 mg g^−1^ flavone *C*-glycosides including 1.933 mg g^−1^ vicenin-2 was obtained. This total yield was 196%, 131%, and 71% more than those obtained using 100% methanol, water, and 70% methanol, respectively.

## 1. Introduction

Orchidaceae, commonly known as the orchid family, is a widespread family of flowering plants comprising up to 35,000 species worldwide [1]. Besides the ornamental value from their exquisite beauty, orchids have historically been recognized to have medicinal values as well [1]. The potentially bioactive phytochemicals produced by Orchidaceae cover a wide range of secondary metabolites including alkaloids, phenanthrenes, flavonoids, and floral fragrances [2]; however, comprehensive and detailed studies on their chemical and biological properties are still very limited. *Cymbidium kanran* is an orchid exclusively distributed in Northeast Asia including China, Japan, and Korea. *C. kanran* has been highly valued as a decorative plant and traditional herbal medicine with beneficial effects on heart, lungs, and gastroenteritis, etc. [3,4]. Chemical profiles of *C. kanran* have not been reported, but several flavone *C*-glycosides including vicenin-2, vitexin 7-*O* glucoside, and isovitexin have been found in a few *Cymbidium* species [5].

**Figure 1:**
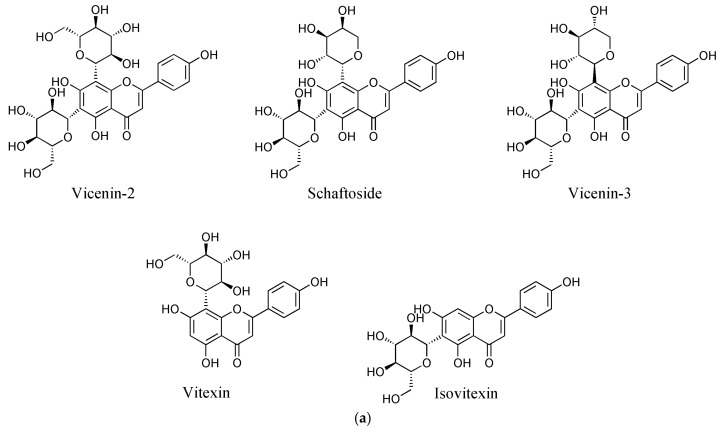

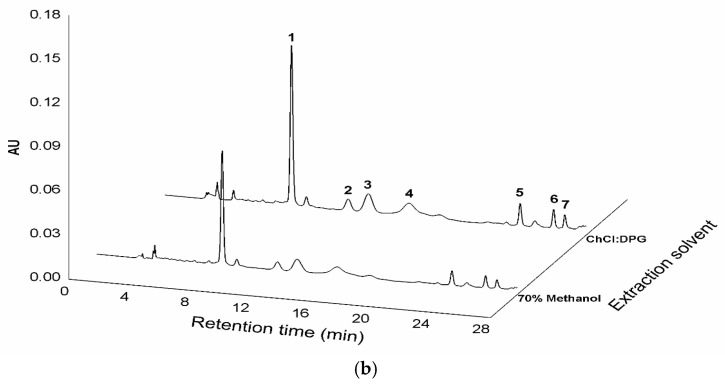
Chemical structures of the flavone *C*-glycosides in *C. kanran* identified in this study (**a**) and chromatograms of the *C. kanran* extracts (**b**). Peak identification: 1, vicenin-2; 2, vicenin-2 isomer; 3, schaftoside isomer; 4, schaftoside; 5, vicenin-3; 6, vitexin; 7, isovitexin.

It is notable that flavone *C*-glycosides are the most common leaf flavonoids, followed by flavonols, in Orchidaceae [1]. Flavone *C*-glycosides, which have a sugar moiety at the C-6 or C-8 position of a flavone A ring, are stable to hydrolysis, unlike *O*-glycosides, and have high biological activities [6]. These have led to a gradual increase in research interest on flavone *C*-glycosides. For example, vicenin-2 is a flavone *C*-glycoside found in diverse plants including Orchidaceae (Figure 1a) [7,8,9] and has shown various biological activities, including anticancer [10], anti-inflammatory [11], and antioxidant effects [12]. As a result, recent studies on vicenin-2 have focused on various aspects from the quality control of herbal medicine [13] to organic synthesis [14,15] to the treatment [10] or prevention [16] of prostate cancer. 

For the chemical characterization and recovery of bioactive compounds from plants, organic solvents with diverse polarities are usually used as the dissolution and extraction media [17]. In particular, polar organic solvents or their aqueous solutions are often used for flavonoid extraction. In conformity with the needs of modern chemistry, replacement of the conventional organic solvents by ecofriendly and safe solvents has been increasingly demanded [18]. In this regard, deep eutectic solvents (DESs) have been suggested as a desirable alternative [19]. DESs are a fluid system composed of two or more components associated via hydrogen bond interactions with a melting point far below either of the individual components [20]. The hydrogen-bond donors (HBDs) include amides, carboxylic acids, alcohols, sugars, and sugar alcohols, while the hydrogen-bond acceptors (HBAs) are usually quaternary ammonium salts. Due to their safety and high solubilization ability regardless of polarity, DESs could serve as a desirable extraction medium [21]. Extraction techniques are also an important factor affecting extraction efficiency. For polyphenol and flavonoid extraction, various extraction methods have been used including heat reflux extraction, microwave-assisted extraction, pressurized liquid extraction, and ultrasound-assisted extraction (UAE) [22]. In particular, UAE is preferable for thermally labile compounds [23] and offers high extraction efficiency by facilitating solvent penetration and tissue disruption [24,25]. 

The aim of this study was to acquire green extracts of *C. kanran* enriched with potentially bioactive compounds at maximized efficiency. For this, first, the chemical profile of flavone *C*-glycosides in *C. kanran* was investigated. Then, a series of DESs were logically designed and synthesized, and a DES with the highest extraction efficiency was sought. Finally, the effects of the extraction conditions based on UAE on the extraction efficiency were assessed, followed by the optimization of the extraction conditions using response surface methodology (RSM).

## 2. Results and Discussion

### 2.1. Chemical Profiling of C. kanran Extracts

For the chemical profiling of *C. kanran*, its extracts were initially prepared in 70% methanol by UAE, which has been suggested as a useful tool to extract or recover bioactive compounds from various sources [26,27,28]. Liquid chromatography-photodiode array (LC-PDA) analysis of the extracts displayed a number of peaks having two λ_max_ values at ~270 nm and ~335 nm between 8 and 28 min (Supplementary Materials Figure S1 and Figure 1b). This type of absorption spectrum is indicative of flavone structures [29]. The weak elution conditions for these peaks indicated that they were likely to be glycosides. Subsequent analysis using ultra-high performance liquid chromatography-quadrupole-time-of-flight mass spectrometry allowed the identification of seven flavone *C*-glycosides (Table 1). The chromatographic peaks 1, 4, 5, 6, and 7 in Figure 1 were confirmed to be vicenin-2, schaftoside, vicenin-3, vitexin, and isovitexin, respectively. Peaks 2 and 3 exhibited the same exact mass values and UV spectra as vicenin-2 and schaftoside, respectively (Table 1), but could not be identified due to difficulty in standard procurement for the candidate compounds. Accordingly, peaks 2 and 3 were putatively named as a vicenin-2 isomer and schaftoside isomer, respectively, and the subsequent studies were conducted by quantifying the seven flavone *C*-glycosides. This is the first report of chemical profiles of flavone *C*-glycosides in a *Cymbidium* species. In addition, the developed LC-PDA method enabled the simultaneous determination of major flavone *C*-glycosides in *C. kanran*.

### 2.2. Preparation of a Series of Deep Eutectic Solvents

Numerous combinations of HBAs and HBDs from renewable, inexpensive, and readily accessible resources are possible [30]. In this study, the HBA was fixed as choline chloride, which usually permits easy synthesis of DESs and therefore is the most common component of DESs [31]. For its counterpart, diol and polyol types were selected as HBDs, because a series of diols and polyols with similar chemical structures were available and because diol- or polyol-based DESs were less viscous than DESs containing carboxylic acids or sugars, therefore easing the solvent handling [20]. 

Specifically, four polyol compounds, from glycerol to maltitol with an increasing number of alcohols, and six diol compounds, from 1,2-ethanediol to 1,6-hexanediol and dipropylene glycol with an increasing carbon chain length, were selected (Table 2). All of the HBD components could form DESs with choline chloride at the ratios described in Table 2 by the heating method, and the resulting solvents are listed in Table 2.

### 2.3. Selection of Deep Eutectic Solvents with High Extraction Efficiency

Although UAE has been very effective with the use of DESs [32,33], the possibility remained that other extraction methods might work better in this study. Thus, three simple extraction methods easily compatible with DESs were tested for comparison including stirring, heating, and heating with stirring methods. As shown in Figure S2, the three methods tested did not differ from each other but were significantly inferior to UAE based on the total extracted amounts of flavone *C*-glycosides. Therefore, UAE was maintained as the extraction method in the following experiments.

Vicenin-2 was predominant among the seven flavone *C*-glycosides in all of the extracts prepared in various solvents (Figure S3). The total yields of aqueous mixtures of organic solvents were higher than those of pure methanol or ethanol, in which yields were much lower than that of water (Figure S3). These results show that the extraction of flavonoid glycosides having both non-polar and polar moieties was favored when using organic solvents with reasonable polarity. 

Table 3 shows that the extraction efficiencies were greatly affected by the DES types, that is, the HBD components. Among the polyol-based DESs, the extraction yields increased as the number of hydroxyl groups of the HBD decreased. In the case of the diol-based DESs, the yields steadily increased with the increasing carbon chain length, e.g., from C_2_ to C_6_. In both cases, the enhanced extraction yields were probably due to the increased hydrophobicity of the HBDs in the DESs. Similar observations have been reported on the effects of carbon length of the cation of ionic liquids on the extraction efficiency [34]. 

The two diol-based DESs, ChCl:Hex and ChCl:DPG, included 1,6-hexanediol and dipropylene glycol, respectively, as the HBDs. They displayed the highest extraction yields. Although the differences between them were not significant, the latter was selected as the final extraction solvent because it exhibited slightly higher values than the former.

### 2.4. Effects of Variables on the Extraction Efficiency for Flavone C-Glycosides

The most important categorical variable influencing the extraction efficiency could be the extraction solvent, which was selected to be ChCl:DPG, as discussed in Section 2.3. Several numerical variables that could be influential are the extraction time, ratio of solvent to sample solids, and water content in the extraction solvent. Therefore, the following three variables were included in a central composite design (CCD) for RSM analysis: (*A*) ultrasonic irradiation time; (*B*) volume of extraction solvent per 100 mg of *C. kanran* powder; and (*C*) content of water in the extraction solvent. They were varied at five levels (−α, −1, 0, +1, +α), and the real ranges tested were as follows: *A*, 0.3–90 min; *B*, 0.5–1.75 mL; *C*, 1.6–100% *w/w*.

The experiments were conducted in random order and showed that the extracted amounts of the individual compounds and their summed amounts varied in a very similar fashion depending on the experimental conditions. Accordingly, the total yields of seven flavone *C*-glycosides were input as the response (*Y*), which yielded the following polynomial quadratic equation in coded values:

$$Y=2.34+0.15A+0.082B-0.29C-0.28\mathrm{AB}+0.22\mathrm{AC}+0.15\mathrm{BC}+0.071A^{2}+0.090B^{2}-0.28C^{2}$$

The model evaluation results based on the analysis of variance (ANOVA) are summarized in Table S1. All of the figures of merit indicated that the established model is valid. 

The equation above and the ANOVA results show that the water content in the extraction solvent (*C* and *C*^2^) was the most influential variable in a negative fashion (*p* < 0.05). That is, lower water contents were preferable for higher extraction yields. This is somewhat consistent with the above observations that the extraction efficiency for flavone *C*-glycosides tended to improve with decreasing polarity of the HBD components. 

In contrast, the extraction time (*A*) had a significant influence in a positive direction (*p* < 0.05), indicating that a longer extraction time could lead to higher yields. The necessity for a longer irradiation time than in previous studies where leaves or skins were used [32,33] might be attributed to the native physical properties of *C. kanran* samples that contained dense tissues such as stalks and roots from the whole plants. The solid-to-liquid ratio (*B*) showed no significant influence. Additionally, information on the interactive effects could be also obtained. A negative interaction was observed between the extraction time and the extractant volume (*p* < 0.05), while the time and the water content had a positive interaction (*p* < 0.05).

### 2.5. Optimization of the Extraction Conditions

A solution was sought for the optimal conditions that could lead to the maximal extraction yields. The resulting conditions involved the extraction of 100 mg of sample powder in 0.53 mL of a mixture of ChCl:DPG and water (74:26, *w/w*) for 86 min of ultrasonic irradiation. When the extraction experiment was actually performed under the optimized conditions, a total yield of 3.441 mg g^−1^ flavone *C*-glycosides (n = 3, 8.46% RSD) was measured, and this was very close to the predicted yield of 3.49 mg g^−1^. Among the total flavone *C*-glycosides extracted was vicenin-2, present at 56% (1.933 mg g^−1^). This total yield is almost as thrice that obtained using 100% methanol and 71% more than the yield obtained using 70% methanol (Figure 1b and Table 3).

## 3. Materials and Methods

### 3.1. Chemicals, Reagents, and Equipment

Whole dried *C. kanran* that had been cultivated in Jeju, Korea was provided by Amorepacific Corporation (Seoul, Korea). They were finely pulverized and kept at −20 °C until analysis. Compounds used for DES preparation are listed in Table 2, and their detailed information is summarized in Table S2. Analytical standards including vicenin-2 (≥98.0%), schaftoside (≥98.0%), and vicenin-3 (≥98.0%) were obtained from Alfa Biotechnology Co., Ltd. (Chengdu, Sichuan, China), while vitexin (95.0%) and isovitexin (98.0%) were purchased from Sigma-Aldrich (St. Louis, MO, USA). Formic acid and trifluoroacetic acid were also from Sigma-Aldrich. HPLC-grade acetonitrile and water were obtained from J.T. Baker (Phillipsburg, NJ, USA). 

An ultrasonic bath (Powersonic 410) and a heating magnetic stirrer were obtained from Hwashin Technology (Seoul, Korea) and VELP Scientifica (Usmate, Italy), respectively.

### 3.2. Preparation of Analytical Standard Solutions and Deep Eutectic Solvents

Each stock solution of vicenin-2, schaftoside, vicenin-3, vitexin, and isovitexin was prepared in methanol at 1 mg mL^−1^ and stored at −20 °C. Standard working solutions were produced in methanol at final concentrations of 2.5, 5, 10, 20, 40, 50, and 80 µg mL^−1^ for vicenin-2; 0.5, 1, 2, 4, 8, 10, and 16 µg mL^−1^ for schaftoside; 0.5, 1, 2, 4, 8, and 10 µg mL^−1^ for vicenin-3 and vitexin; and 0.25, 0.5, 1, 2, 4, and 5 µg mL^−1^ for isovitexin. These were used for validation of the quantitative method assessing linearity, precision, and accuracy. DESs were synthesized using a heating method as previously described [32]. For the solvent selection procedure, DESs were mixed with water to produce 85% *w/w* aqueous mixtures for easy handling [35].

### 3.3. Ultra-High Performance Liquid Chromatography-Quadrupole-Time-of-Flight Mass Spectrometry Analysis of C. kanran Extracts

The experimental conditions were slightly modified from a previous study [36]. The stationary phase was an ACQUITY UPLC BEH C18 column (50 mm × 2.1 mm, 1.7 µm) from Waters (Milford, MA, USA), and a linear gradient elution was conducted using a mobile phase consisting of (A) 0.1% formic acid in water and (B) 0.1% formic acid in acetonitrile: 0–5 min, 1–10% B; 5–8 min, 10–25% B; 8–10 min 25–45% B; 10–16 min 45–75% B; 16–18 min, 75–100% B; 18–21 min, 100% B. Parameters of the mass spectrometer were set as follows: capillary voltage, 1.9 kV; sample cone, 40 V; source temperature, 100 °C; desolvation gas (nitrogen), 1000 L h^−1^. The high collision energy ramps ranged from 10 to 20 V. Samples were diluted 10-fold with water and filtered through a 0.2-µm membrane filter (Whatman, Piscataway, NJ, USA) before injection.

### 3.4. Liquid Chromatography-Photodiode Array Detection Analysis of C. kanran Extracts

A Waters LC system equipped with a Waters separations module (series 2695) and a Waters photodiode array detector (series 996) was used. Empower software was used for system operation and data management. The detection wavelength was 335 nm. Chromatographic separation was achieved on a Gemini C_18_ 110 Å column (5 µm, 4.6 mm × 250 mm) from Phenomenex (Torrance, CA, USA) at a flow rate of 1.0 mL min^−1^ at room temperature. The mobile phase consisted of 0.1% trifluoroacetic acid in water (A) and 0.1% trifluoroacetic acid in acetonitrile (B), and the linear gradient elution was as follows: 0–5 min, 15.5% B; 5–10 min, 15.5–12.6% B; 10–15 min, 12.6–13.2% B; 15–20 min, 13.2–18.5% B; 20–25 min, 18.5–23.0% B; 25–27 min, 23.0% B; 27–28 min, 23.0–100.0% B. The system was returned to the initial conditions and equilibrated for 10 min before subsequent injections. Samples diluted in water were membrane-filtered prior to injection.

The quantification method was validated as previously described [32]. The calibration curves for vicenin-2, schaftoside, vicenin-3, vitexin, and isovitexin were linear (r^2^ ≥ 0.9963), precise (≤12.6% RSD), and accurate (90.8–112.6%) within the tested ranges (please see supplementary materials for detailed results).

### 3.5. Extraction of Flavone C-Glycosides from C. kanran

The following conditions were used for UAE before the extraction conditions were optimized. One hundred mg of the dried *C. kanran* powder were mixed in 1 mL of extraction solvent (water, methanol, ethanol, 70% aqueous methanol, 70% aqueous ethanol, or DESs). After being vortexed briefly, the mixture was irradiated at ambient temperature under the maximum power (~500 W) without thermal control for 45 min. The bath temperature tended to rise with increasing irradiation time; it was approximately 40 °C after extraction. After centrifugation at 12,300× *g* for 20 min, the cleared supernatant was removed and diluted with water for chromatographic analysis. After condition optimization, the extraction was conducted under the conditions described in Section 2.5.

Stirring, heating, and heating with stirring methods were tested in comparison to the UAE method as follows: 100 mg of *C. kanran* powder were extracted in 1 mL of 70% methanol for 45 min by stirring (600 rpm, room temperature), heating (60 °C), or heating with stirring (600 rpm, 60 °C). Each extract was analyzed by LC-PDA.

Extraction yields were expressed as the individual or total amount (mg) of extracted flavone *C*-glycosides per mass (g) of *C. kanran* powder (mg g^−1^).

### 3.6. Experimental Design and Statistical Analysis

RSM based on a CCD was conducted using the Design-Expert Ver. 8.0 (Statease Inc., Minneapolis, MN, USA). Further statistical analysis was performed using GraphPad Prism 5.01 for Windows (GraphPad Software, San Diego, CA, USA) as previously described [32].

## 4. Conclusions

In this study, the chemical profile of *C. kanran* was investigated for the first time, and it was found that *C. kanran* is rich in flavone *C*-glycosides, especially vicenin-2, the most prevalent one that comprised more than 50% of the total flavone *C*-glycosides. Ten different DESs were logically designed by varying either the number of alcohol groups in polyol type solvents or the length of the carbon chain in diol type solvents. The solvent extraction efficiency for the flavone *C*-glycosides improved with fewer alcohol groups or longer carbon chain lengths in the HBD component, which is probably because the non-polar property from the HBD compartment was needed for efficient interaction with the hydrophobic flavone moiety. The RSM-based investigation of several variables for the extraction suggested that lower water content in the extraction solvent and longer extraction time during UAE were desirable for higher extraction yields. The final optimized conditions resulted in the extraction of a total of 3.441 mg g^−1^ flavone *C*-glycosides including 1.933 mg g^−1^ vicenin-2; this total yield is 196%, 131%, and 71% more than those obtained using 100% methanol, water, and 70% methanol, respectively. This study showed that the potentially bioactive flavone *C*-glycosides including vicenin-2 could be extracted in DES with maximal efficiency under the optimized conditions. 

## Figures and Tables

**Table 1:** Identification of the major flavone *C*-glycosides in *C. kanran.*

No. ^a^	Compound	λ_max_^b^	MW ^c^	Molecular Formula	Theoretical		Measured		Mass Error ^d^	
					[M + H]^+^	[M − H]^−^	[M + H]^+^	[M − H]^−^	[M + H]^+^	[M − H]^−^
1	Vicenin-2	334	594.1584	C_27_H_30_O_15_	595.1663	593.1506	595.1655	593.1509	−1.3	0.5
2	Vicenin-2 isomer	333	594.1584	C_27_H_30_O_15_	595.1663	593.1506	595.1658	593.1501	−0.8	−0.8
3	Schaftoside isomer	335	564.1479	C_26_H_28_O_14_	565.1557	563.1401	565.1546	563.1392	−1.9	−1.6
4	Schaftoside	336	564.1479	C_26_H_28_O_14_	565.1557	563.1401	565.1542	563.1411	−2.7	1.8
5	Vicenin-3	334	564.1479	C_26_H_28_O_14_	565.1557	563.1401	565.1541	563.1404	−2.8	0.5
6	Vitexin	336	432.1056	C_21_H_20_O_10_	433.1135	431.0978	433.1136	431.0976	0.2	−0.5
7	Isovitexin	336	432.1056	C_21_H_20_O_10_	433.1135	431.0978	433.1122	431.0981	−3.0	0.7

**^a^** Peak identification number in Figure 1; **^b^** nm; **^c^** g mol^−1^; **^d^** ppm.

**Table 2:** List of the choline chloride-based deep eutectic solvents (DESs) synthesized in this study.

Abbreviation	Hydrogen Bond Acceptor	Hydrogen Bond Donor	Molar Ratio
ChCl:Gly	Choline chloride	Glycerol	1:4
ChCl:Xyl		Xylitol	1:1
ChCl:Sor		d-sorbitol	1:1
ChCl:Mal		Maltitol	1:1
ChCl:Eth		1,2-Ethanediol	1:4
ChCl:Prop		1,3-Propanediol	1:4
ChCl:But		1,4-Butanediol	1:4
ChCl:Pent		1,5-Pentanediol	1:4
ChCl:Hex		1,6-Hexanediol	1:4
ChCl:DPG		Dipropylene glycol	1:4

**Table 3:** Extraction yields for flavone *C*-glycosides of the tested solvents.

Extraction Solvent	Extracted Amount ^a^ (Mean ± SD, n = 3)							
	Vicenin-2	Vicenin-2 Isomer	Schaftoside Isomer	Schaftoside	Vicenin-3	Vitexin	Isovitexin	Summed Amount
Water	0.940 (±0.002)	0.115 (±0.002)	0.157 (±0.007)	0.174 (±0.007)	0.072 (±0.002)	0.023 (±0.002)	0.005 (±0.000)	1.486 ***^, b^ (±0.016)
100% Methanol	0.623 (±0.022)	0.090 (±0.003)	0.156 (±0.004)	0.150 (±0.002)	0.066 (±0.001)	0.048 (±0.001)	0.025 (±0.000)	1.158 *** (±0.034)
70% Methanol	1.130 (±0.006)	0.158 (±0.006)	0.262 (±0.007)	0.258 (±0.012)	0.107 (±0.001)	0.057 (±0.001)	0.029 (±0.000)	2.001 *** (±0.017)
70% Ethanol	1.186 (±0.015)	0.169 (±0.002)	0.281 (±0.006)	0.264 (±0.009)	0.116 (±0.001)	0.060 (±0.000)	0.031 (±0.000)	2.107 *** (±0.019)
ChCl:Gly	1.153 (±0.061)	0.151 (±0.001)	0.258 (±0.010)	0.225 (±0.005)	0.102 (±0.006)	0.052 (±0.004)	0.025 (±0.002)	1.966 *** (±0.092)
ChCl:Xyl	0.964 (±0.031)	0.127 (±0.005)	0.208 (±0.003)	0.192 (±0.004)	0.082 (±0.002)	0.041 (±0.001)	0.019 (±0.000)	1.633 *** (±0.040)
ChCl:Sor	0.859 (±0.033)	0.111 (±0.010)	0.178 (±0.012)	0.164 (±0.015)	0.073 (±0.004)	0.035 (±0.002)	0.015 (±0.000)	1.435 *** (±0.075)
ChCl:Mal	0.774 (±0.062)	0.093 (±0.004)	0.162 (±0.008)	0.144 (±0.018)	0.063 (±0.004)	0.031 (±0.001)	0.014 (±0.000)	1.281 *** (±0.085)
ChCl:Eth	1.210 (±0.068)	0.161 (±0.006)	0.244 (±0.002)	0.219 (±0.002)	0.110 (±0.006)	0.057 (±0.003)	0.027 (±0.000)	2.028 *** (±0.086)
ChCl:Prop	1.275 (±0.121)	0.175 (±0.017)	0.288 (±0.027)	0.272 (±0.034)	0.120 (±0.010)	0.065 (±0.005)	0.032 (±0.003)	2.227 *** (±0.220)
ChCl:But	1.324 (±0.104)	0.184 (±0.012)	0.298 (±0.025)	0.283 (±0.029)	0.128 (±0.011)	0.069 (±0.006)	0.034 (±0.003)	2.320 *** (±0.194)
ChCl:Pent	1.460 (±0.058)	0.193 (±0.002)	0.328 (±0.001)	0.296 (±0.021)	0.141 (±0.006)	0.076 (±0.002)	0.040 (±0.002)	2.534 ** (±0.049)
ChCl:Hex	1.533 (±0.009)	0.197 (±0.013)	0.339 (±0.006)	0.325 (±0.005)	0.151 (±0.002)	0.079 (±0.001)	0.040 (±0.000)	2.664 (±0.004)
ChCl:DPG	1.652 (±0.052)	0.233 (±0.003)	0.370 (±0.021)	0.347 (±0.026)	0.162 (±0.005)	0.087 (±0.003)	0.045 (±0.000)	2.896 (±0.108)
Optimized conditions	1.933 (±0.158)	0.277 (±0.025)	0.441 (±0.039)	0.452 (±0.039)	0.188 (±0.014)	0.100 (±0.010)	0.050 (±0.004)	3.441 (±0.291)

^a^ mg g^−1^; ^b^ statistical difference in comparison to ChCl:DPG was indicated with ** (*p* < 0.01) and *** (*p* < 0.001).

## Supplementary Materials

The following are available online. Results S1: Validation results of the LC-PDA method for flavone *C*-glycosides; Figure S1: a two dimensional chromatogram from the LC-PDA analysis of *C. kanran* extracts obtained in 70% aqueous methanol; Figure S2: extraction efficiency of heating, stirring, heating with stirring, and UAE methods using 70% aqueous methanol; Figure S3: overlaid chromatograms of the *C. kanran* extracts obtained in water, methanol, ethanol, 70% methanol, 70% ethanol, and 10 different DESs. Table S1: ANOVA results of the established model; Table S2: compounds used for the preparation of deep eutectic solvents.
