# Supplementary material for: Identification of Major Flavone C-Glycosides and Their Optimized Extraction from Cymbidium kanran Using Deep Eutectic Solvents
Source: Molecules. 2017 Nov 18;22(11):2006. doi: 10.3390/molecules22112006 (PMC6150217; doi:10.3390/molecules22112006)
Supplement: Supplementary file 1 [file molecules-22-02006-s001.pdf]

Supplementary Materials

Identification of major flavone *C*-glycosides and their optimized extraction from *Cymbidium kanran* using deep eutectic solvents

Kyung Min Jeong [1], Misuk Yang [2], Yan Jin [1], Eun Mi Kim [1], Jaeyoung Ko [2,*], and Jeongmi Lee [1,*]

[1] School of Pharmacy, Sungkyunkwan University, Jangan-gu, Suwon 16419, Gyeonggi-do, Korea
[2] Amorepacific Research and Development Center, Giheung-gu, Yongin 17074, Gyeonggi-do, Korea

* Correspondence: jaeyoungko@amorepacific.com (J.K.); jlee0610@skku.edu (J.L.); Tel.: +82-31-280-5928 (J.K.); +82-31-290-7784 (J.L.)

Results S1. Validation results of the LC-PDA method for flavone *C*-glycosides

The calibration curve for each compound including vicenin-2, schaftoside, vicenin-3, vitexin, and isovitexin was plotted as peak area versus concentration of each standard. Linearity of the calibration curve was evaluated based on the coefficient of determination (r^2). The resulting linear regression equations and linear ranges were as follows: $y = 32329x + 6791$ ($r^2 = 0.9969$) for vicenin-2; $y = 48223x + 319.2$ ($r^2 = 0.9969$) for schaftoside; $y = 46507x + 1133$ ($r^2 = 0.9965$) for vicenin-3; $y = 66822x + 1885$ ($r^2 = 0.9963$) for vitexin; $y = 86332x + 1725$ ($r^2 = 0.9971$) for isovitexin.

The intra-day and inter-day precisions were less than 12.6% RSD and 10.1% RSD, respectively. The intra-day and inter-day accuracies obtained were 90.8-112.6% (n=3), and 95.2-105.6% (n=3×3) in all tested QC samples, respectively.

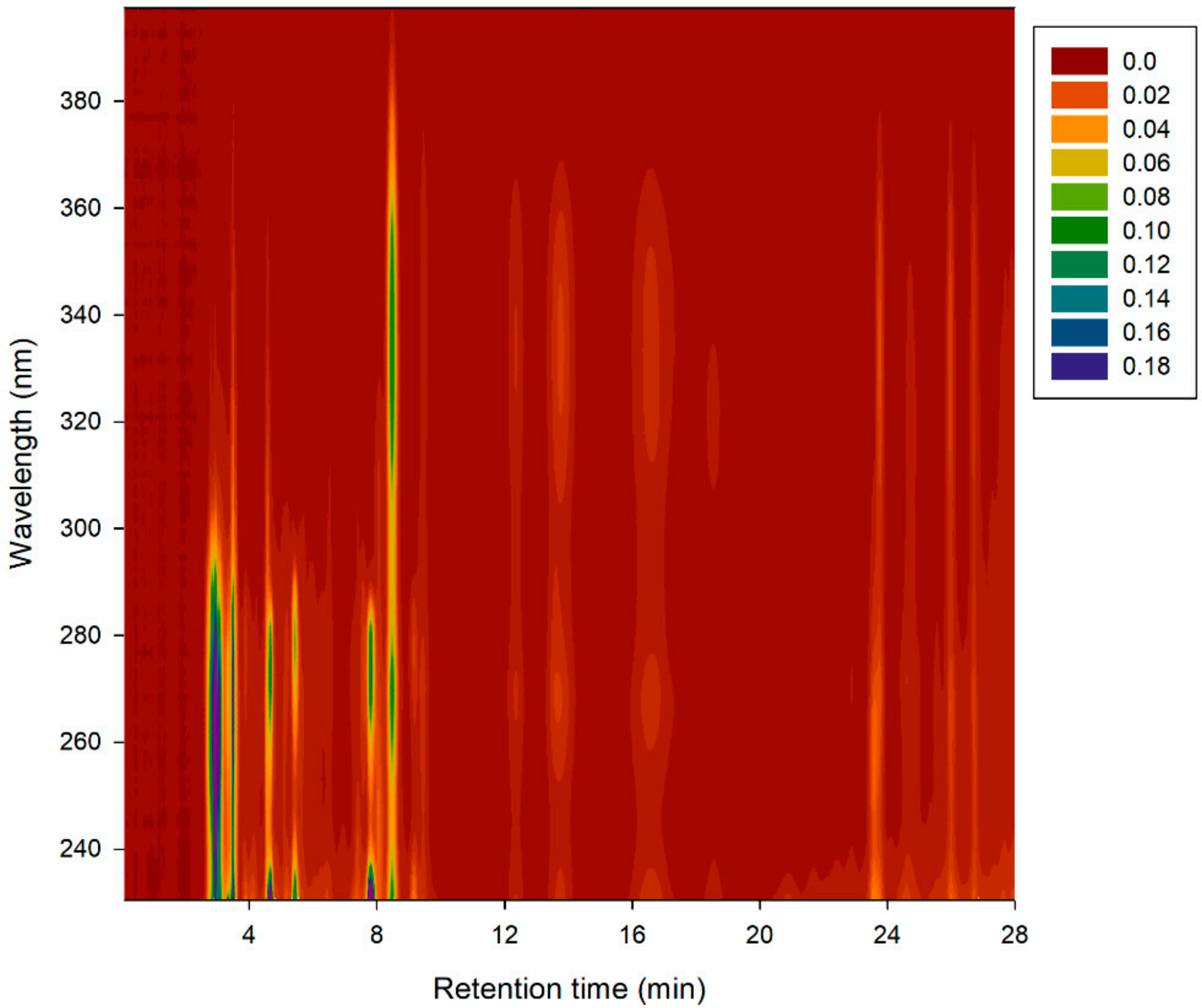

Figure S1. A two dimensional chromatogram from the LC-PDA analysis of *C. kanran* extracts obtained in 70% aqueous methanol.

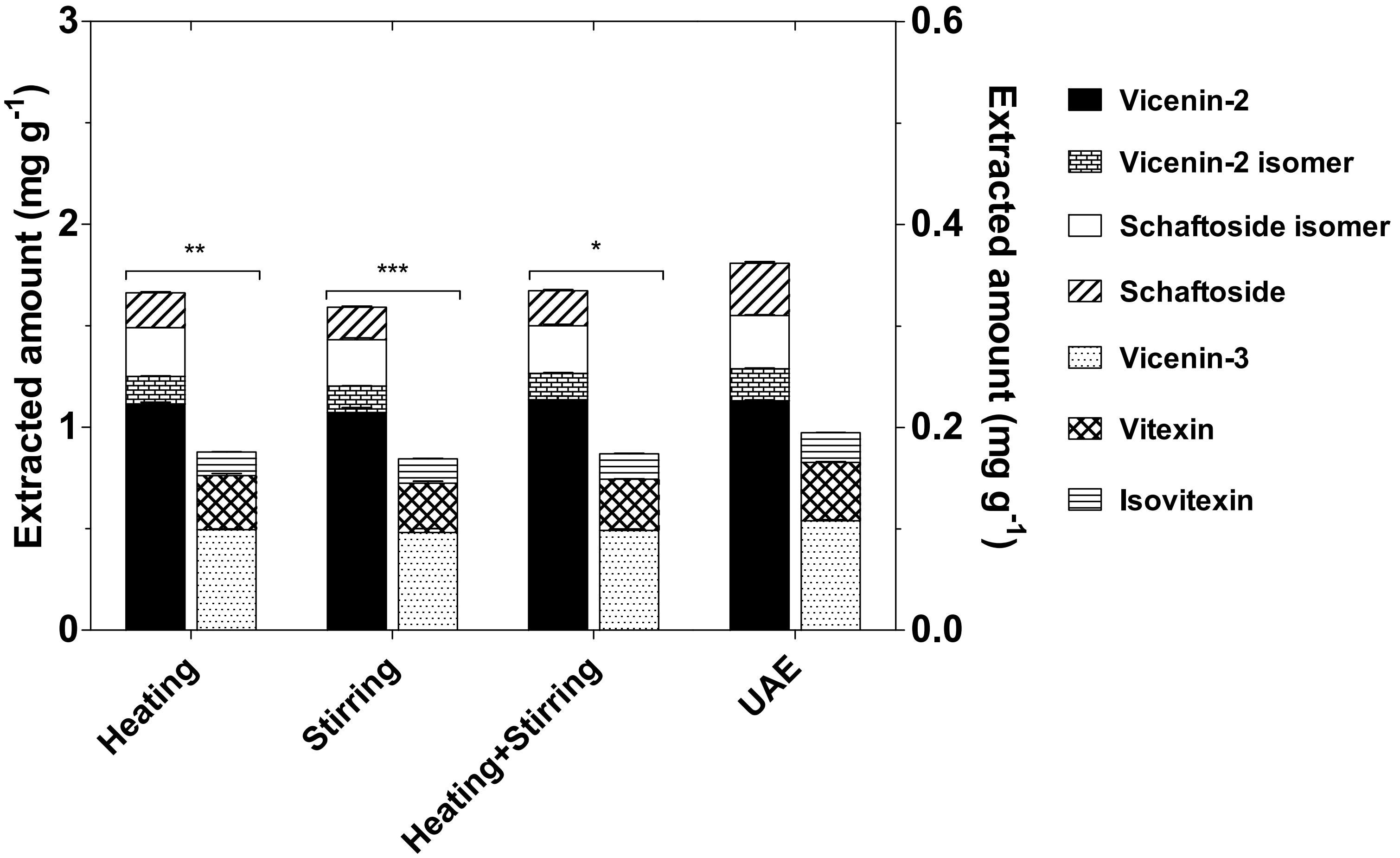

Figure S2. Extraction efficiency of heating, stirring, heating with stirring, and UAE methods using 70% aqueous methanol. Extracted amounts of the total flavone *C*-glycosides of the UAE method were compared with those of the other extraction methods. * ($p < 0.05$), ** ($p < 0.01$), and *** ($p < 0.001$). Error bars represent the SEM (n = 3).

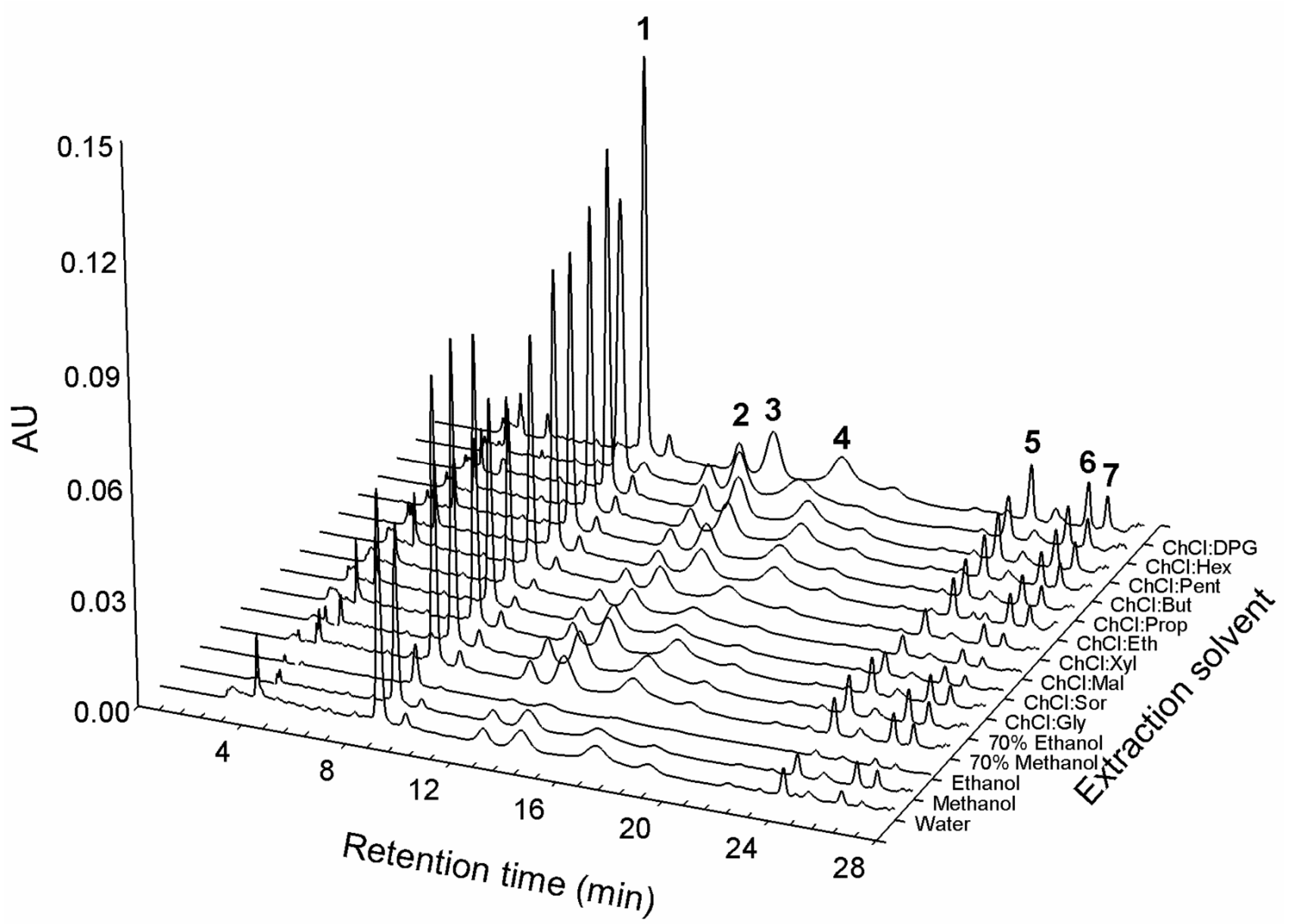

Figure S3. Overlaid chromatograms of the *C. kanran* extracts obtained in water, methanol, ethanol, 70% methanol, 70% ethanol, and 10 different DESs. Peak identification; 1, vicenin-2; 2, vicenin-2 isomer; 3, schaftoside isomer; 4, schaftoside; 5, vicenin-3; 6, vitexin; 7, isovitexin.

Table S1. ANOVA results of the established model.

Source	Sum of squares	Degree of freedom	Mean square	*F* value	Prob > *F*
Block	1.77	2	0.88		
Model	4.20	9	0.47	9.51	0.0021
A	0.30	1	0.30	6.21	0.0374
B	0.092	1	0.092	1.89	0.2069
C	1.14	1	1.14	23.26	0.0013
AB	0.61	1	0.61	12.47	0.0077
AC	0.39	1	0.39	7.98	0.0223
BC	0.18	1	0.18	3.75	0.0887
A^2	0.073	1	0.073	1.48	0.2578
B^2	0.12	1	0.12	2.40	0.1599
C^2	1.15	1	1.15	23.54	0.0013
Residual	0.39	8	0.049		
Lack of fit	0.35	5	0.071	5.61	0.0933
Pure error	0.038	3	0.013		
R^2	0.9146				

Table S2. Compounds used for the preparation of deep eutectic solvents.

Compound	Purity	Source
Choline chloride	≥98.0%	
Glycerol	≥99.5%	
D-sorbitol	≥99.5%	
Maltitol	≥98.0%	
Xylitol	≥99.0%	
1,2-Ethanediol	≥99.8%	Sigma-Aldrich (St. Louis, MO, USA)
1,3-Propanediol	≥98.0%	
1,4-Butanediol	≥99.0%	
1,5-Pentanediol	≥97.0%	
1,6-Hexanediol	≥99.0%	
Dipropylene glycol	≥99.0%	
